# (5*Z*)-5-(2-Hy­droxy­benzyl­idene)-3-(4-methyl­phen­yl)-2-sulfanyl­idene-1,3-thia­zolidin-4-one

**DOI:** 10.1107/S1600536812021630

**Published:** 2012-05-19

**Authors:** Durre Shahwar, M. Nawaz Tahir, Misbah Kashif, Afifa Saeed, Sana Bukhari

**Affiliations:** a Department of Chemistry, Government College University, Lahore, Pakistan; b University of Sargodha, Department of Physics, Sargodha, Pakistan

## Abstract

In the title compound, C_17_H_13_NO_2_S_2_, the dihedral angles between the 2-sulfanyl­idene-1,3-thia­zolidin-4-one group and the pendant toluene and 2-hy­droxy­benzene rings are 74.62 (6) and 8.73 (12)°, respectively. An intra­molecular C—H⋯S inter­action occurs. In the crystal, inversion dimers linked by pairs of O—H⋯O hydrogen bonds generate *R*_2_^2^(16) loops. This link is reinforced by a pair of C—H⋯O hydrogen bonds. The dimers are connected by weak C—H⋯S inter­actions.

## Related literature

For related structures and further synthetic details, see: Shahwar *et al.* (2009**a* ▶,b* ▶). For graph-set notation, see: Bernstein *et al.* (1995 ▶).
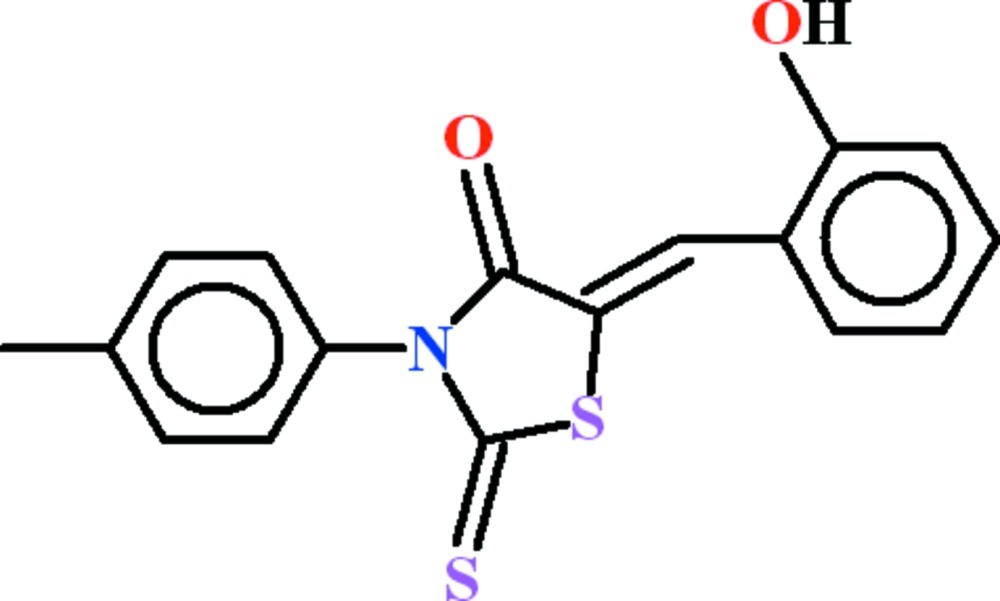


## Experimental

### 

#### Crystal data

C_17_H_13_NO_2_S_2_
*M* *_r_* = 327.40
Monoclinic, 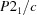

*a* = 13.8258 (6) Å
*b* = 5.4278 (3) Å
*c* = 21.0715 (9) Å
β = 101.857 (3)°
*V* = 1547.54 (13) Å^3^
*Z* = 4
Mo *K*α radiation
μ = 0.35 mm^−1^
*T* = 296 K
0.35 × 0.15 × 0.13 mm

#### Data collection

Bruker Kappa APEXII CCD diffractometer
Absorption correction: multi-scan (*SADABS*; Bruker, 2005 ▶) *T*_min_ = 0.945, *T*_max_ = 0.965
10652 measured reflections
2801 independent reflections
1473 reflections with *I* > 2σ(*I*)
*R*_int_ = 0.069

#### Refinement

*R*[*F* ^2^ > 2σ(*F* ^2^)] = 0.047
*wR*(*F* ^2^) = 0.101
*S* = 0.93
2801 reflections
201 parameters
H-atom parameters constrained
Δρ_max_ = 0.19 e Å^−3^
Δρ_min_ = −0.26 e Å^−3^

### 

Data collection: *APEX2* (Bruker, 2009 ▶); cell refinement: *SAINT* (Bruker, 2009 ▶); data reduction: *SAINT*; program(s) used to solve structure: *SHELXS97* (Sheldrick, 2008 ▶); program(s) used to refine structure: *SHELXL97* (Sheldrick, 2008 ▶); molecular graphics: *ORTEP-3 for Windows* (Farrugia, 1997 ▶) and *PLATON* (Spek, 2009 ▶); software used to prepare material for publication: *WinGX* (Farrugia, 1999 ▶) and *PLATON*.

## Supplementary Material

Crystal structure: contains datablock(s) global, I. DOI: 10.1107/S1600536812021630/hb6792sup1.cif

Structure factors: contains datablock(s) I. DOI: 10.1107/S1600536812021630/hb6792Isup2.hkl

Supplementary material file. DOI: 10.1107/S1600536812021630/hb6792Isup3.cml

Additional supplementary materials:  crystallographic information; 3D view; checkCIF report

## Figures and Tables

**Table 1:** Hydrogen-bond geometry (Å, °)

*D*—H⋯*A*	*D*—H	H⋯*A*	*D*⋯*A*	*D*—H⋯*A*
O2—H2*A*⋯O1^i^	0.82	1.92	2.712 (3)	162
C6—H6⋯S2^ii^	0.93	2.84	3.736 (4)	163
C11—H11⋯O2^i^	0.93	2.38	3.294 (4)	167
C13—H13⋯S1	0.93	2.48	3.194 (3)	133

Symmetry codes: (i) 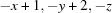; (ii) 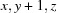.

**Fig. 1.**
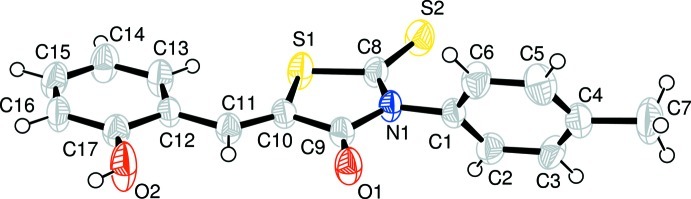
View of the title compound with displacement ellipsoids drawn at the 50% probability level.

**Fig. 2.**
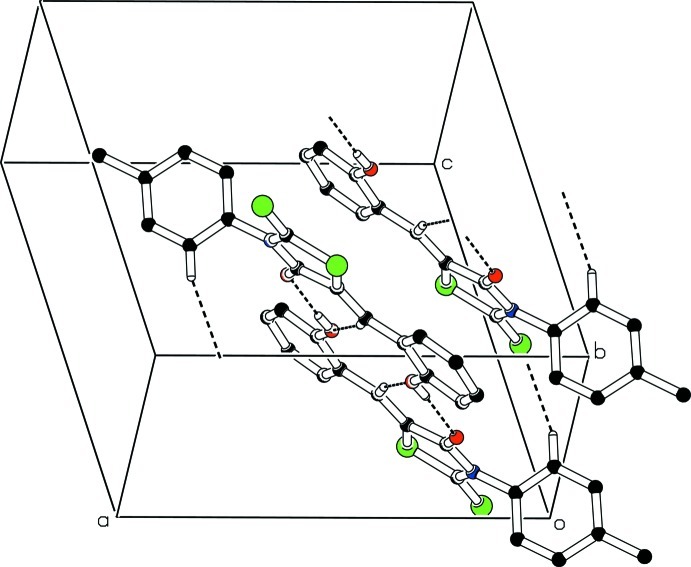
The partial packing, showing that molecules form dimers with R22(16) and other ring motifs.

## supplementary crystallographic information

### Comment

In the search for new enzyme inhibitors, a series of rohdanine derivatives were prepared and their crystal structures have been reported such as (5*Z*)-5-(2-hydroxybenzylidene)-3-phenyl-2-sulfanylidene-1,3-thiazolidin-4-one (Shahwar *et al.*, 2009*a*) and (5*Z*)-5-(2-methylbenzylidene)-3-phenyl-2-sulfanylidene-1,3-thiazolidin-4-one (Shahwar *et al.*, 2009*b*) which are related to the title compound, (I), (Fig. 1).

In (I), the toluene group A (C1–C7), group B (N1/C8/S1/C10/C9/O1/S2) of 2-sulfanylidene-1,3-thiazolidin-4-one and group C (C11–C17/O2) of 2-methylphenol are planar with r. m. s. deviation of 0.0246 Å, 0.0186 Å and 0.0175 Å, respectively. The dihedral angle between A/B, A/C and B/C is 74.62 (6), 70.16 (7) and 8.73 (12)°, respectively. The molecules are dimerized due to strong O—H···O type of H-bonding with *R*_2_^2^(16) (Bernstein *et al.*, 1995) ring motifs (Table 1, Fig. 2). *R*_2_^2^(7) ring motifs are formed within the dimers when weak H-boding of C—H···O type is considered. The dimers are interlinked due to C—H···S type of H-bondings (Table 1, Fig. 2).

### Experimental

3-(4-methylphenyl)-2-sulfanylidene-1,3-thiazolidin-4-one (0.419 g, 0.2 mol) (prepared according to the method: Shahwar *et al.* 2009*a*), 2-hydroxybenzaldehyde (0.244 g, 0.2 mol) and K_2_CO_3_ (0.553 g, 0.4 mol) were dissolved in distilled water (10 ml) and continuously stirred for 24 h. The mixture was neutralized with 5% HCl and the crude product obtained was recrysrallized in ethylacetate to get the yellow needles of (I).

### Refinement

The H-atoms were positioned geometrically (C—H = 0.93–0.96 Å, O—H = 0.82 Å) and refined as riding with *U*_iso_(H) = *xU*_eq_(C, O), where *x* = 1.5 for methyl groups and *x* = 1.2 for other H atoms.

### Crystal data

**Table:**

C_17_H_13_NO_2_S_2_	*F*(000) = 680
*M**_r_* = 327.40	*D*_x_ = 1.405 Mg m^−^^3^
Monoclinic, *P*2_1_/*c*	Mo *K*α radiation, λ = 0.71073 Å
Hall symbol: -P 2ybc	Cell parameters from 1473 reflections
*a* = 13.8258 (6) Å	θ = 3.0–25.3°
*b* = 5.4278 (3) Å	µ = 0.35 mm^−^^1^
*c* = 21.0715 (9) Å	*T* = 296 K
β = 101.857 (3)°	Needle, yellow
*V* = 1547.54 (13) Å^3^	0.35 × 0.15 × 0.13 mm
*Z* = 4	

### Data collection

**Table:**

Bruker Kappa APEXII CCD diffractometer	2801 independent reflections
Radiation source: fine-focus sealed tube	1473 reflections with *I* > 2σ(*I*)
Graphite monochromator	*R*_int_ = 0.069
Detector resolution: 8.20 pixels mm^-1^	θ_max_ = 25.3°, θ_min_ = 3.0°
ω scans	*h* = −16→16
Absorption correction: multi-scan (*SADABS*; Bruker, 2005)	*k* = −6→6
*T*_min_ = 0.945, *T*_max_ = 0.965	*l* = −25→25
10652 measured reflections	

### Refinement

**Table:**

Refinement on *F*^2^	Primary atom site location: structure-invariant direct methods
Least-squares matrix: full	Secondary atom site location: difference Fourier map
*R*[*F*^2^ > 2σ(*F*^2^)] = 0.047	Hydrogen site location: inferred from neighbouring sites
*wR*(*F*^2^) = 0.101	H-atom parameters constrained
*S* = 0.93	*w* = 1/[σ^2^(*F*_o_^2^) + (0.0385*P*)^2^] where *P* = (*F*_o_^2^ + 2*F*_c_^2^)/3
2801 reflections	(Δ/σ)_max_ < 0.001
201 parameters	Δρ_max_ = 0.19 e Å^−^^3^
0 restraints	Δρ_min_ = −0.26 e Å^−^^3^

### Special details

**Table:**

Geometry. Bond distances, angles *etc*. have been calculated using the rounded fractional coordinates. All su's are estimated from the variances of the (full) variance-covariance matrix. The cell e.s.d.'s are taken into account in the estimation of distances, angles and torsion angles
Refinement. Refinement of *F*^2^ against ALL reflections. The weighted *R*-factor *wR* and goodness of fit *S* are based on *F*^2^, conventional *R*-factors *R* are based on *F*, with *F* set to zero for negative *F*^2^. The threshold expression of *F*^2^ > σ(*F*^2^) is used only for calculating *R*-factors(gt) *etc*. and is not relevant to the choice of reflections for refinement. *R*-factors based on *F*^2^ are statistically about twice as large as those based on *F*, and *R*- factors based on ALL data will be even larger.

### Fractional atomic coordinates and isotropic or equivalent isotropic displacement parameters (Å^2^)

**Table:**

	*x*	*y*	*z*	*U*_iso_*/*U*_eq_	
S1	0.32730 (6)	0.24641 (18)	0.08942 (4)	0.0555 (3)	
S2	0.14244 (6)	−0.0283 (2)	0.04718 (4)	0.0633 (4)	
O1	0.28996 (14)	0.6397 (4)	−0.06441 (10)	0.0510 (8)	
O2	0.57469 (16)	1.0037 (5)	0.06997 (10)	0.0773 (10)	
N1	0.21224 (16)	0.3302 (5)	−0.02022 (11)	0.0383 (9)	
C1	0.1268 (2)	0.3173 (6)	−0.07351 (14)	0.0387 (11)	
C2	0.1162 (2)	0.1227 (7)	−0.11500 (14)	0.0486 (13)	
C3	0.0316 (2)	0.1030 (7)	−0.16312 (14)	0.0531 (14)	
C4	−0.0423 (2)	0.2766 (7)	−0.16993 (15)	0.0486 (13)	
C5	−0.0286 (2)	0.4723 (7)	−0.12792 (17)	0.0598 (14)	
C6	0.0553 (2)	0.4943 (7)	−0.07940 (15)	0.0534 (12)	
C7	−0.1379 (2)	0.2463 (7)	−0.21949 (16)	0.0805 (16)	
C8	0.2200 (2)	0.1799 (6)	0.03366 (14)	0.0436 (11)	
C9	0.2879 (2)	0.5003 (7)	−0.01944 (15)	0.0404 (11)	
C10	0.3612 (2)	0.4829 (6)	0.04263 (13)	0.0408 (10)	
C11	0.4379 (2)	0.6400 (6)	0.05800 (13)	0.0418 (11)	
C12	0.5128 (2)	0.6614 (6)	0.11718 (13)	0.0391 (11)	
C13	0.5208 (2)	0.5059 (7)	0.17093 (14)	0.0530 (14)	
C14	0.5919 (2)	0.5422 (7)	0.22614 (15)	0.0592 (14)	
C15	0.6561 (2)	0.7355 (7)	0.22960 (15)	0.0564 (14)	
C16	0.6513 (2)	0.8925 (7)	0.17828 (15)	0.0520 (13)	
C17	0.5808 (2)	0.8546 (6)	0.12202 (14)	0.0446 (11)	
H2	0.16539	0.00361	−0.11109	0.0582*	
H2A	0.62157	1.09941	0.07623	0.0928*	
H3	0.02469	−0.03032	−0.19141	0.0637*	
H5	−0.07693	0.59368	−0.13213	0.0718*	
H6	0.06290	0.62792	−0.05118	0.0641*	
H7A	−0.12882	0.12593	−0.25115	0.1208*	
H7B	−0.15615	0.40109	−0.24058	0.1208*	
H7C	−0.18916	0.19251	−0.19813	0.1208*	
H11	0.44377	0.75226	0.02568	0.0501*	
H13	0.47704	0.37487	0.16928	0.0640*	
H14	0.59639	0.43528	0.26110	0.0709*	
H15	0.70355	0.76061	0.26727	0.0677*	
H16	0.69516	1.02395	0.18109	0.0625*	

### Atomic displacement parameters (Å^2^)

**Table:**

	*U*^11^	*U*^22^	*U*^33^	*U*^12^	*U*^13^	*U*^23^
S1	0.0601 (5)	0.0596 (7)	0.0403 (5)	−0.0257 (5)	−0.0047 (4)	0.0102 (5)
S2	0.0689 (6)	0.0671 (8)	0.0527 (5)	−0.0349 (6)	0.0096 (4)	0.0025 (5)
O1	0.0484 (13)	0.0539 (16)	0.0449 (13)	−0.0174 (12)	−0.0037 (11)	0.0132 (13)
O2	0.0795 (18)	0.087 (2)	0.0509 (14)	−0.0543 (16)	−0.0205 (12)	0.0243 (16)
N1	0.0362 (14)	0.0385 (18)	0.0362 (14)	−0.0112 (14)	−0.0015 (12)	0.0016 (14)
C1	0.0347 (17)	0.041 (2)	0.0385 (18)	−0.0079 (18)	0.0031 (15)	0.0005 (18)
C2	0.0429 (19)	0.053 (3)	0.0473 (19)	0.0042 (19)	0.0032 (17)	−0.006 (2)
C3	0.057 (2)	0.056 (3)	0.0422 (19)	−0.011 (2)	0.0009 (18)	−0.0158 (19)
C4	0.0417 (19)	0.056 (3)	0.0441 (19)	−0.011 (2)	−0.0002 (16)	0.011 (2)
C5	0.049 (2)	0.050 (3)	0.075 (2)	0.006 (2)	0.000 (2)	0.007 (2)
C6	0.053 (2)	0.043 (2)	0.060 (2)	−0.005 (2)	0.0019 (18)	−0.013 (2)
C7	0.055 (2)	0.112 (4)	0.061 (2)	−0.014 (2)	−0.0196 (18)	0.012 (3)
C8	0.0447 (18)	0.044 (2)	0.0401 (18)	−0.0132 (18)	0.0042 (15)	−0.0036 (18)
C9	0.0370 (18)	0.040 (2)	0.0431 (19)	−0.0070 (18)	0.0054 (16)	−0.0045 (19)
C10	0.0372 (17)	0.047 (2)	0.0363 (17)	−0.0089 (18)	0.0032 (14)	0.0034 (18)
C11	0.0417 (18)	0.044 (2)	0.0374 (17)	−0.0101 (18)	0.0026 (15)	0.0061 (17)
C12	0.0345 (17)	0.042 (2)	0.0379 (18)	−0.0092 (18)	0.0007 (15)	0.0006 (18)
C13	0.052 (2)	0.053 (3)	0.047 (2)	−0.014 (2)	−0.0059 (17)	0.008 (2)
C14	0.064 (2)	0.061 (3)	0.044 (2)	−0.012 (2)	−0.0091 (18)	0.014 (2)
C15	0.056 (2)	0.066 (3)	0.0393 (19)	−0.008 (2)	−0.0089 (17)	−0.002 (2)
C16	0.0414 (18)	0.062 (3)	0.046 (2)	−0.0162 (19)	−0.0066 (16)	−0.002 (2)
C17	0.0452 (19)	0.047 (2)	0.0386 (18)	−0.0076 (19)	0.0016 (16)	0.0036 (19)

### Geometric parameters (Å, º)

**Table:**

S1—C8	1.731 (3)	C12—C17	1.398 (4)
S1—C10	1.741 (3)	C12—C13	1.399 (4)
S2—C8	1.623 (3)	C13—C14	1.374 (4)
O1—C9	1.218 (4)	C14—C15	1.367 (5)
O2—C17	1.352 (4)	C15—C16	1.368 (5)
O2—H2A	0.8200	C16—C17	1.387 (4)
N1—C1	1.455 (4)	C2—H2	0.9300
N1—C9	1.393 (4)	C3—H3	0.9300
N1—C8	1.384 (4)	C5—H5	0.9300
C1—C2	1.360 (5)	C6—H6	0.9300
C1—C6	1.366 (5)	C7—H7A	0.9600
C2—C3	1.386 (4)	C7—H7B	0.9600
C3—C4	1.376 (5)	C7—H7C	0.9600
C4—C5	1.371 (5)	C11—H11	0.9300
C4—C7	1.516 (4)	C13—H13	0.9300
C5—C6	1.385 (4)	C14—H14	0.9300
C9—C10	1.484 (4)	C15—H15	0.9300
C10—C11	1.347 (4)	C16—H16	0.9300
C11—C12	1.453 (4)		
			
C8—S1—C10	93.57 (14)	C14—C15—C16	120.7 (3)
C17—O2—H2A	109.00	C15—C16—C17	119.7 (3)
C1—N1—C8	121.2 (2)	O2—C17—C16	121.4 (3)
C1—N1—C9	122.0 (2)	C12—C17—C16	121.2 (3)
C8—N1—C9	116.7 (2)	O2—C17—C12	117.5 (3)
N1—C1—C6	119.6 (3)	C1—C2—H2	120.00
C2—C1—C6	120.5 (3)	C3—C2—H2	120.00
N1—C1—C2	119.8 (3)	C2—C3—H3	119.00
C1—C2—C3	119.6 (3)	C4—C3—H3	119.00
C2—C3—C4	121.4 (3)	C4—C5—H5	119.00
C3—C4—C7	121.4 (3)	C6—C5—H5	119.00
C5—C4—C7	121.0 (3)	C1—C6—H6	120.00
C3—C4—C5	117.5 (3)	C5—C6—H6	120.00
C4—C5—C6	121.8 (3)	C4—C7—H7A	109.00
C1—C6—C5	119.2 (3)	C4—C7—H7B	109.00
S1—C8—S2	122.09 (18)	C4—C7—H7C	109.00
S1—C8—N1	110.2 (2)	H7A—C7—H7B	109.00
S2—C8—N1	127.7 (2)	H7A—C7—H7C	109.00
O1—C9—N1	122.7 (3)	H7B—C7—H7C	110.00
O1—C9—C10	127.2 (3)	C10—C11—H11	115.00
N1—C9—C10	110.1 (3)	C12—C11—H11	115.00
S1—C10—C11	128.4 (2)	C12—C13—H13	119.00
C9—C10—C11	122.2 (3)	C14—C13—H13	119.00
S1—C10—C9	109.4 (2)	C13—C14—H14	120.00
C10—C11—C12	130.1 (3)	C15—C14—H14	120.00
C11—C12—C17	118.2 (3)	C14—C15—H15	120.00
C13—C12—C17	117.0 (3)	C16—C15—H15	120.00
C11—C12—C13	124.8 (3)	C15—C16—H16	120.00
C12—C13—C14	121.5 (3)	C17—C16—H16	120.00
C13—C14—C15	119.9 (3)		
			
C10—S1—C8—S2	−177.0 (2)	C3—C4—C5—C6	−1.2 (5)
C10—S1—C8—N1	1.8 (2)	C7—C4—C5—C6	175.5 (3)
C8—S1—C10—C9	−2.5 (2)	C4—C5—C6—C1	0.5 (5)
C8—S1—C10—C11	174.7 (3)	O1—C9—C10—S1	−177.6 (3)
C8—N1—C1—C2	−73.7 (4)	O1—C9—C10—C11	5.0 (5)
C8—N1—C1—C6	102.9 (4)	N1—C9—C10—S1	2.6 (3)
C9—N1—C1—C2	109.1 (4)	N1—C9—C10—C11	−174.8 (3)
C9—N1—C1—C6	−74.3 (4)	S1—C10—C11—C12	−1.0 (5)
C1—N1—C8—S1	−177.9 (2)	C9—C10—C11—C12	175.9 (3)
C1—N1—C8—S2	0.9 (4)	C10—C11—C12—C13	2.8 (5)
C9—N1—C8—S1	−0.6 (3)	C10—C11—C12—C17	−175.5 (3)
C9—N1—C8—S2	178.2 (2)	C11—C12—C13—C14	−177.9 (3)
C1—N1—C9—O1	−3.8 (5)	C17—C12—C13—C14	0.4 (5)
C1—N1—C9—C10	176.0 (3)	C11—C12—C17—O2	−2.6 (4)
C8—N1—C9—O1	178.9 (3)	C11—C12—C17—C16	177.0 (3)
C8—N1—C9—C10	−1.4 (4)	C13—C12—C17—O2	179.0 (3)
N1—C1—C2—C3	175.8 (3)	C13—C12—C17—C16	−1.5 (4)
C6—C1—C2—C3	−0.7 (5)	C12—C13—C14—C15	0.7 (5)
N1—C1—C6—C5	−176.1 (3)	C13—C14—C15—C16	−0.8 (5)
C2—C1—C6—C5	0.5 (5)	C14—C15—C16—C17	−0.3 (5)
C1—C2—C3—C4	0.0 (5)	C15—C16—C17—O2	−179.0 (3)
C2—C3—C4—C5	1.0 (5)	C15—C16—C17—C12	1.4 (5)
C2—C3—C4—C7	−175.7 (3)		

### Hydrogen-bond geometry (Å, º)

**Table:**

*D*—H···*A*	*D*—H	H···*A*	*D*···*A*	*D*—H···*A*
O2—H2*A*···O1^i^	0.82	1.92	2.712 (3)	162
C6—H6···S2^ii^	0.93	2.84	3.736 (4)	163
C11—H11···O2^i^	0.93	2.38	3.294 (4)	167
C13—H13···S1	0.93	2.48	3.194 (3)	133

Symmetry codes: (i) −*x*+1, −*y*+2, −*z*; (ii) *x*, *y*+1, *z*.
